# Introduction to artificial intelligence in ultrasound imaging in obstetrics and gynecology

**DOI:** 10.1002/uog.22122

**Published:** 2020-10-01

**Authors:** L. Drukker, J. A. Noble, A. T. Papageorghiou

**Affiliations:** ^1^ Nuffield Department of Women's & Reproductive Health University of Oxford, John Radcliffe Hospital Oxford UK; ^2^ Institute of Biomedical Engineering University of Oxford Oxford UK

## Abstract

Artificial intelligence (AI) uses data and algorithms to aim to draw conclusions that are as good as, or even better than, those drawn by humans. AI is already part of our daily life; it is behind face recognition technology, speech recognition in virtual assistants (such as Amazon Alexa, Apple's Siri, Google Assistant and Microsoft Cortana) and self‐driving cars. AI software has been able to beat world champions in chess, Go and recently even Poker. Relevant to our community, it is a prominent source of innovation in healthcare, already helping to develop new drugs, support clinical decisions and provide quality assurance in radiology. The list of medical image‐analysis AI applications with USA Food and Drug Administration or European Union (soon to fall under European Union Medical Device Regulation) approval is growing rapidly and covers diverse clinical needs, such as detection of arrhythmia using a smartwatch or automatic triage of critical imaging studies to the top of the radiologist's worklist. Deep learning, a leading tool of AI, performs particularly well in image pattern recognition and, therefore, can be of great benefit to doctors who rely heavily on images, such as sonologists, radiographers and pathologists. Although obstetric and gynecological ultrasound are two of the most commonly performed imaging studies, AI has had little impact on this field so far. Nevertheless, there is huge potential for AI to assist in repetitive ultrasound tasks, such as automatically identifying good‐quality acquisitions and providing instant quality assurance. For this potential to thrive, interdisciplinary communication between AI developers and ultrasound professionals is necessary. In this article, we explore the fundamentals of medical imaging AI, from theory to applicability, and introduce some key terms to medical professionals in the field of ultrasound. We believe that wider knowledge of AI will help accelerate its integration into healthcare. © 2020 The Authors. Ultrasound in Obstetrics & Gynecology published by John Wiley & Sons Ltd on behalf of the International Society of Ultrasound in Obstetrics and Gynecology.

## Introduction

Artificial intelligence (AI) is described as the ability of a computer program to perform processes associated with human intelligence, such as reasoning, learning, adaptation, sensory understanding and interaction^1^. In his seminal paper published in 1950^2^, Alan Turing introduced a test (now called ‘the Turing test’) in which, if an evaluator cannot distinguish whether intelligent behavior is exhibited by a machine or a human, the machine is said to have passed the test^2^. John McCarthy coined the term ‘artificial intelligence’ soon after^3^. The Journal of Artificial Intelligence commenced publication in 1970, but it took several years for computing power to match theoretical possibilities and allow development of modern algorithms.

In simple terms, traditional computational algorithms are software programs that follow a sequence of rules and perform an identical function every time, such as an electronic calculator: ‘if this is the input, then that is the output’. In contrast, an AI algorithm learns the rules (function) from training data (input) presented to it. Major milestones in the history of AI include the Deep Blue computer outmatching the world chess champion, Gary Kasparov, in 1997 and AlphaGo defeating one of the best players (ranked 9‐dan) of the ancient Chinese game of Go, Lee Sedol, in 2016^4^.

Both chess and Go are games that require strategy, foresight and logic, all of which are qualities typically attributed to human intelligence. Go is considered much more difficult for computers than chess, because it involves far more possible moves (approximately 8 million choices for three moves as opposed to 40 000 for chess). The victory in Go represents the progress in computational algorithms, improved computing infrastructure and access to enormous amounts of data. The same evolution has led to several widely popularized AI consumer applications, including autocomplete on Google search, virtual assistants (such as Alexa, Cortana, Google Home and Siri), personalized shopping recommendations, the emergence of automatic self‐driving cars and face recognition (for instance, searching by a face in Google photos).

In clinical medicine, the interest (and recent hype) in AI technologies stems from their potential to transform healthcare by deriving new and important insights from the vast amount of digital data generated during delivery of healthcare. Promising medical AI applications are emerging in the areas of screening^5^, ^6^, prediction^7^, ^8^, ^9^, triage^10^, ^11^, diagnosis^12^, ^13^, drug development^14^, ^15^, treatment^16^, ^17^, monitoring^18^ and imaging interpretation^19^, ^20^. Several original studies published in this Journal have used AI methodology to evaluate adnexal masses^21^, the risk of lymph node metastases in endometrial cancer^22^, pelvic organ function^23^, ^24^ and breast lesions^25^, ^26^, ^27^, assess aneuploidy risk^28^, predict fetal lung maturity^29^, perinatal outcome^30^, shoulder dystocia^31^ and brain damage^32^, estimate gestational age in late pregnancy^33^ and classify standard fetal brain images as normal or abnormal^34^ (Table 1). The number of AI‐related papers is increasing; at the 29^th^ World Congress of the International Society of Ultrasound in Obstetrics and Gynecology (ISUOG) in 2019, there were 14 abstracts specifically mentioning AI, in comparison to a total of 13 abstracts in the preceding six ISUOG World Congresses (2013–2018).

**Table 1:** Examples of reported and expected future artificial intelligence (AI) applications in obstetric and gynecological ultrasound

AI application	Description	Clinical utility
Probe guidance	Operator is guided how to manipulate probe to acquire fetal biometric plane	Facilitate sonographer training; basic scanning can be performed by non‐expert (e.g. general practitioner)
Fetal biometric plane finder	Standard fetal biometric planes are automatically acquired, measured and stored	Reduce repetitive caliper adjustment clicks; reduce operator bias; instant quality control
Anomaly scan completeness	Anomaly scan checklist of mandatory planes is populated automatically	Ensure completeness of imaging and that all parts of anatomy are checked
Anomaly highlighting	Unusual fetal findings are identified in a standard plane	Highlight suspected abnormal finding; assist sonographer with referral decision
Cyst classification	Ovarian cysts are classified according to IOTA criteria	Improve consistency; reduce likelihood of error
Lung scans for Ob/Gyn	Ob/Gyn experts are taught how to perform lung ultrasound in patients with COVID19	Reduce learning curve

COVID19, coronavirus disease 2019;

IOTA, International Ovarian Tumor Analysis.

As with any scientific discipline, the AI scientific community uses technical language and terminology that can be difficult to understand for those outside the area. This in addition to the rapid advancement in the field can make it challenging for other disciplines to keep abreast of developments in AI. Indeed, one of the key concerns that has been expressed regarding AI in medicine is that there are relatively few interdisciplinary professionals who work at the interface of AI and medicine and can ‘translate’ between the two^35^. A recent review of 250 AI papers emphasized the need for greater collaboration between computational scientists and medical professionals to generate more scientifically sound and impactful work integrating knowledge from both domains^36^.

**Figure 1:**
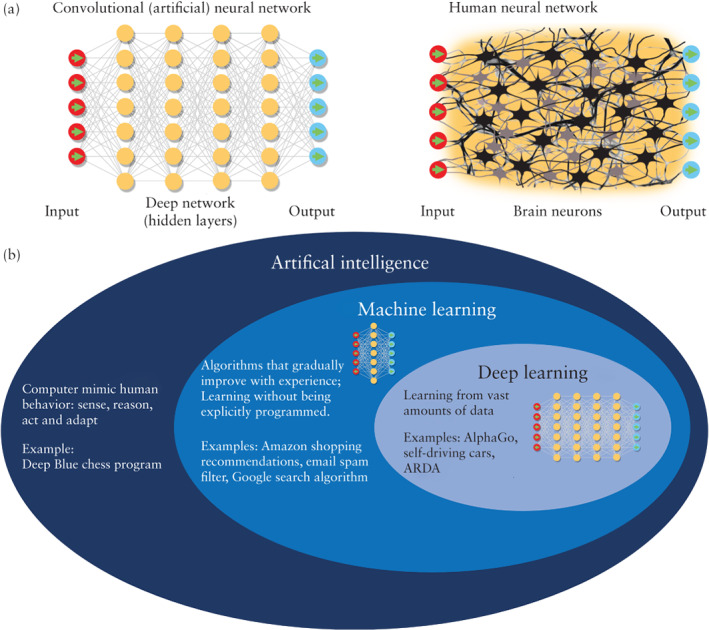
Graphic representation of artificial intelligence. (a) Human neural network architecture and its resemblance to a deep artificial neural network. (b) Relationship between artificial intelligence, machine learning and deep learning. ARDA, automated retinal disease assessment (Appendix S1).

To contribute to this discussion, this article aims to explain key AI‐related concepts and terms to clinicians in the field of ultrasound in obstetrics and gynecology. For simplicity, we use the general term ‘artificial intelligence (AI)’, which is commonly used by others in the field, although most articles referring to AI in clinical medicine are based on deep learning, a subset of AI (Box 1, Figure 1). It is also important to appreciate that relatively few AI‐based ultrasound applications have advanced the whole way from academic concept to clinical application and commercialization. Therefore, we also use examples from radiology, being our closest sister field.

## Artificial intelligence and medical imaging

The current interest in AI in medical imaging stems from major advances in deep learning‐based ‘computer vision’ over the past decade. The field of computer vision concerns computers that interpret and understand the visual world. Within computer vision, object recognition (‘what can I see in this image?’) is a key task which can be posed as an image classification problem. Researchers in this field use ‘challenge’ datasets to benchmark the progress in accuracy of image classification. One such challenge dataset, called the ImageNet project, is a database of more than 14 million images of every day (non‐medical) objects that have been labeled by humans into more than 20 000 categories. This large database was first made available to the scientific community in 2010 to train algorithms for image classification. In 2015, the ImageNet annual competition reached a milestone when the error rate of automatic classification of images dropped below 5%, which is the average human error rate (Figure S1)^17^. This was largely due to advances in deep learning, the branch of AI that learns from large amounts of data.

Deep learning excels in pattern recognition and we believe that medical professions which rely on imaging will be the first to see the benefits of this tool (Appendix S1). One of the largest driving forces behind AI in medical imaging is the enormous amount of digital data generated around the world that may be useful in training algorithms. As of May 2020, there are more than 50 deep learning‐based imaging applications^37^ approved by the USA Food and Drug Administration (FDA) or the European Union, spanning across most imaging modalities, including X‐ray, computerized tomography (CT), magnetic resonance imaging, retinal optical coherence tomography and ultrasound. Approved AI applications are designed to provide increased productivity by performing automated screening, assisting in diagnosis or prioritizing a radiology study that needs to be ‘at the top of the list’. Applications include identification of cerebrovascular accidents, diabetic retinopathy, skeletal fractures, cancer, pulmonary embolism and pneumothorax^37^. Recently, the first ultrasound AI application that guides the user received FDA approval; the software uses AI to help the user capture images of acceptable diagnostic quality during adult echocardiography^38^. The market of AI applications in medical imaging alone is forecasted to top $2 billion by 2023^39^.

Box 1 **Glossary of commonly used artificial intelligence terms**

Artificial intelligence (AI) refers to a machine or software performing tasks that would ordinarily require human brainpower to accomplish, such as making sense of spoken language, learning behaviors or solving problems (Figure 1). This means that an AI program can learn from real‐world data as well as experience, and encompasses the capacity to improve its performance given more data. Nevertheless, there is no accepted definition of AI, and therefore, the term is often misused^71^. AI can be broken down into general AI, which is human‐like intelligence (i.e. ability to think, learn, reason) and narrow AI, which is the ability to perform a specific task (i.e. image detection, translation, chess‐playing).

Convolutional neural networks (CNNs), also known as artificial neural networks, are computational algorithms inspired by the biological neural networks that constitute animal brains and consist of multilayered artificial neurons (Figure 1). A CNN is displayed as a system of hidden connections between input and output. CNNs have the ability to determine the relationship between *input* (such as brain computerized tomography (CT)) and *labels* (presence or absence of hemorrhage). This is in contrast to traditional software, in which predetermined logic rules set the output to specific stimuli. In reality, there is little resemblance to human neurons.

Black box is the term often used to describe the process occurring inside the hidden layers of CNNs. For example, a new AI product is launched aimed at detecting intracranial hemorrhage. When this software reads a CT scan that has signs of intracranial hemorrhage, it will correctly output the result of evidence of intracranial hemorrhage to the care team, yet it may not report why it reached this conclusion. There is an ongoing effort aimed at providing *‘explainability’* to AI, to report the ‘how’ in addition to the result (*Explainable AI*).

Explainable AI is an emerging subfield of AI that attempts to explain how *black box* decisions of AI systems are made. Explainable AI aims to understand the key steps involved in making computational decisions. This should theoretically allow decisions taken by an algorithm to be understood by end‐users.

Model, application or algorithm are all terms used interchangeably for the ready‐to‐use AI software/product.

Machine learning is a branch of AI, defined by the ability to learn from data without being explicitly programmed (Figure 1). Machine learning can be understood as a statistical method that gradually improves as it is exposed to more data, by extracting patterns from data.

Deep learning is a branch of *machine learning* (Figure 1). In deep learning, the input and output are connected by multiple layers of hidden connections, also known as CNNs. Deep learning involves learning from vast amounts of data and performs especially well in pattern recognition within data; therefore, it can be particularly helpful in medical imaging. Deep learning is usually divided into two major classes:

1) Supervised learning, in which *labeled* (*annotated*) data are used as an input to a CNN (Appendix S1). For example, to build an application detecting brain hemorrhage on a CT scan, the CNN is first *trained* using *labeled* data, i.e. normal scans and scans with hemorrhage labeled with the correct diagnosis by a radiologist (label = hemorrhage present/absent). Following training using the *training dataset*, evaluation of the CNN is carried out using a *test dataset* that contains *unlabeled data*; these are new CT scans (not contained in the *training dataset*) with and without hemorrhage that do not have *labels*. The CNN outputs its prediction based on the test data. After validation of the prediction accuracy, the model is ready to use. For instance, the final model is a software that can read a brain CT scan (*input* = CT scan) and decide whether or not intracranial hemorrhage is present or absent (*output* = yes/no hemorrhage).

2) Unsupervised learning is a training process that does not require labeling. This saves the time‐consuming, labor‐intensive and expensive human labeling process. In the intracranial hemorrhage example, the learning input would be CT scans of patients with and without hemorrhage that are not labeled (i.e. the machine is never told if bleeding is absent or present). The CNN will learn by clustering scans that look similar to one another (learn from similarities and differences), which should result in classifying images to either hemorrhage or no hemorrhage.

Big data: In order to achieve good performance, supervised AI applications require a large volume of labeled training data (usually images) from which to learn. Establishing a clinically relevant, well‐curated dataset that can be used to *train* an algorithm can be a very time‐intensive process, and the accuracy of such curation determines the quality of the derived model.

What about ultrasound? Ultrasound AI software needs to fit into the workflow differently from, for example, in the analysis of a CT scan; in ultrasound, real‐time analysis at the point of acquisition is ideally needed, while in CT, automated reading is only needed at the end of the examination. Compared to the image acquisition and analysis abilities of a sonologist, no known current AI method is generic enough to be applied on a wide range of tasks (e.g. an AI application designed for the second trimester is unlikely to be applicable to the first‐trimester scan). For each ultrasound task, there are several image acquisition and analysis capabilities that can be met by an AI application, including classification (‘what objects are present in this image?’), segmentation (‘where are the organ boundaries?’), navigation (‘how can I acquire the optimal image?’), quality assessment (‘is this image fit for purpose to make a diagnosis?’) and diagnosis (‘what is wrong with the imaged object?’). Active academic research and emerging examples of AI‐assisted applications for ultrasound include plane‐finding (navigation) and automated quantification for analysis of the breast, prostate, liver and heart^40^, ^41^, ^42^. In obstetric and gynecological ultrasound, promising workload‐changing advancements include automatic detection of standard planes and quality assurance in fetal ultrasound^43^, ^44^, ^45^, detection of endometrial thickness in gynecology^46^ and automatic classification of ovarian cysts (Table 1).

## Challenges

The introduction of AI into clinical practice offers many potential benefits, but there are also many challenges and uncertainties that may raise concerns.

The impact of AI on jobs is among the most widely discussed concerns^47^, ^48^, ^49^. Major technological advances frequently impact the job market, and the current wave of AI‐based automation is no exception. However, this does not automatically imply technological unemployment; rather, it may trigger a transformation in the way we work, resulting in professional realignment. AI can enhance both the value and the professional satisfaction of sonographers and maternal–fetal medicine experts by reducing the time needed for routine tasks and allowing more time to perform tasks that add value and influence patient care^49^, ^50^. An important advantage that machines have over humans is reproducibility: machines retain absolute consistency over time whereas the performance of a clinician varies depending on many factors, such as years of experience, fatigue or simple distractions, such as a late‐running clinic or a ringing phone. Additionally, an AI application has higher capacity, theoretically being able to read thousands of scans, while a radiographer reads 50–100 scans per day^49^. Evidence in the literature suggests that the first wave of AI applications is likely to constitute assistive technology, taking over repetitive tasks to improve consistency, such as reading radiographs^51^. Specifically in ultrasound, automation will assist in shortening the total scan duration by removing the need for some of the tiresome or ‘simple’ repetitive tasks, such as acquiring standard planes, annotation or adjustment of calipers (Table 1). This may allow more time to analyze additional scan planes or to communicate the results to patients^52^. Automation should also be seen in the context of a global shortage of imaging experts, including sonographers and radiographers, while demand for diagnostic imaging is rising^53^.

Applicability is another concern relating to the implementation of AI in clinical medicine. Imaging features alone are often not sufficient to determine diagnosis and management. Consider, for instance, an AI application developed to report on ovarian cysts that is designed to produce a binary outcome of malignant features being absent or present based on an ovarian imaging training dataset. Clinicians also take into account the clinical context, including many factors such as age, menopausal status and familial risk factors, when making a diagnosis. While it could be argued that the clinician may be biased by clinical information, this example highlights the importance of understanding when an AI solution is applicable and when it is not. AI models can account only for information ‘seen’ during training, so in this example, non‐imaging clinical information is not taken into account by the AI model. Hence, an important emerging area of healthcare AI research focuses on building AI models that integrate imaging and electronic health record data for ‘personalized diagnostic imaging’^54^, ^55^.

Another fear, which is largely unwarranted, relates to adaptable systems, which are AI applications that continue to learn, adapt and optimize based on new data and hence may jeopardize the application's safety. Regulatory bodies, including the FDA, currently approve only AI applications with models that have ‘locked’ parameters^56^, ^57^. This means that all current AI applications are static models that can no longer adapt, and therefore, the approved product does not change over time.

The ‘black box’ design of AI applications is attractive at one level, as there is no need to understand how the complex non‐linear optimization works, but is also a source of concern as clinicians want to understand any associated bias and likely modes of failure. Most AI models are derived by using ‘supervised learning’, meaning that the model learns from data annotated by humans (Box 1). Since human involvement can potentially introduce bias to the learning process, the resulting model could also be biased. Understanding model bias is an important aspect of AI model design and an active area of research^58^. For example, as operators seem to be at risk of expected‐value bias when acquiring fetal biometry measurements, an algorithm training to measure standard biometric planes by supervised learning might end up having a built‐in bias when automatically calculating fetal biometry^59^. To better understand AI model bias, as well as to provide insights into how AI algorithms make decisions, ‘explainable AI’ is an emerging subfield of AI research aiming to demystify the black box design.

Deep learning excels in pattern recognition, but it is important to recognize that most methods are supervised (training data are manually annotated). Manual annotation is resource‐intensive and is often subjective. Most academic publications use data annotated a single time by one or more human annotators, which means that the derived model will be biased by (or skewed towards) the human annotator's preferred method of annotation. If, instead, each image is annotated by multiple humans, then there need to be rules about how to agree on consensus if their annotations differ. There is no one way to do this. Thus, one can appreciate that the process of annotation and subsequent data cleaning is both resource‐intensive and determines the success of model performance. Furthermore, traditional deep‐learning methods require a considerable volume of data to build accurate models, which are not always available. There are some ways to address this limitation which are the subject of current deep learning in medical imaging research. These include using pre‐trained models, which essentially allow initialization of the parameters of a new model with those of a model built for another problem, and allowing new data to update model parameters. Another issue deep‐learning scientists have to consider is deployability, as traditional deep‐learning models can have millions of parameters and take up lots of computer memory. Models can be reduced in size empirically, and there is an emerging area of interest in designing small deep neural networks, such as MobileNet and SESNet, as the backbone for deployable AI application models.

Unfortunately, there are high expectations of AI applications which have yet to be backed up by wide‐scale convincing multicenter clinical studies and, when appropriate, randomized clinical trials. An interesting overview of the current standards of AI research in medical imaging is provided in a recent publication^60^. Indeed, most of the reported AI applications to date use data from a single site and focus on algorithm performance rather than looking at clinical utility or health economics^60^. It is particularly challenging to assess an AI model when the accuracy of a human expert for the same task is difficult to determine or is unknown^60^. It is important to appreciate that healthcare AI is an emerging technology and, as such, it will take time to determine the best ways to validate and regulate AI applications. Towards this goal, a recent multinational academic report addressing both medical and non‐medical AI systems, entitled ‘Toward Trustworthy AI Development: Mechanisms for Supporting Verifiable Claims’^61^, provides a list of measures and mechanisms for AI developers and regulatory bodies to ensure responsible AI development. Among the recommendations, the report calls for introduction of third‐party auditing of AI systems, creating a system for reporting AI incidents and encouraging researchers in academia to verify claims made by industry.

No discussion about AI would be complete without mentioning ethics^62^. Recently, the classic theoretical ‘trolley problem’ experiment was applied to self‐driving cars, as part of an online experimental platform designed to explore human perspective on moral decisions made by autonomous vehicles^63^. The question is whose safety should be prioritized in the event of an accident. Essentially, the problem asks: if your car brakes suddenly fail as you speed toward a crowded crosswalk, and you are confronted with the dilemma to veer right and crash into an old man or veer left and crash into a concrete wall and kill the car driver and passengers, what would you choose? Now, what if instead of an old man, it was a woman pushing a stroller or a homeless person crossing the road? Human drivers who are badly injured or die in a car crash cannot report whether they were faced with a dilemma. However, self‐driving cars can be programmed to act in a certain way. Similarly, the use of AI‐based solutions may raise several moral questions in medicine^64^, ^65^: would we trust computers to screen for disease, prioritize treatment, diagnose, treat, discharge? Would we let a fully automated AI‐based solution choose the patient to occupy the only available intensive care unit bed?

Ethical concerns also surround the issue of privacy^65^, ^66^. Developing AI applications typically requires a large volume of data about patients and their diagnoses. Such personal data are usually collected by health authorities or hospitals. Under what conditions (if any) should hospitals be allowed to share patient data with developers of AI solutions, who may be commercial entities? If healthcare data are completely anonymized, does a patient need to expressly consent to their use for such improvements in healthcare? These questions, which relate to data governance and privacy, are not unique to healthcare AI and are currently being debated widely by regulators, policy‐makers, technologists and technology end‐users (including the public). An emerging technology area, called privacy‐enhancing technologies, may offer data‐sharing and analysis options to reduce some of the current barriers and concerns.

Potential professional liability for physicians using AI is another challenge^67^. Should hospitals and doctors be accountable for decisions that an AI application makes? Information provided by an AI application may be used to inform clinical management, diagnosis or treatment. However, algorithms, like humans, can err. Let us suppose that an AI algorithm classifies an ovarian cyst as most likely benign and recommends follow‐up imaging in 6 months according to the standard of care; at the next appointment, the patient is diagnosed with metastatic ovarian cancer and retrospective image review suggests that the ‘cyst’ may have had malignant features previously. This raises the question: who is liable when AI‐based diagnosis is incorrect? Questions of this kind are currently being considered by regulators, in consultation with legal professionals, medical professionals and AI developers in the industry.

## Research in context

As we begin to see more interdisciplinary research related to AI in clinical medicine, difficulties arise when readers and reviewers with a clinical background attempt to critically assess the methodology of scientific AI papers in a field that is, for now, largely unfamiliar to many medical professionals. How can the clinical research community ensure that highly technical aspects of a scientific work have been conducted and presented correctly^68^? Ultrasound professionals understand the full meaning of ‘sonographer with 10 years of experience’ or ‘images were reviewed by two specialists’, but may struggle with descriptions such as ‘A feed‐forward network of neurons consisting of a number of layers that are connected to each other was built.’^28^ or ‘To train the model, we first provided the sample input, x, to the first layer and acquired the best parameters (W, b) and activated the first hidden layer, y, and then utilized y to predict the second layer.’^30^. When assessing the clinical effectiveness and legitimacy of scientific work for publication, several crucial questions should be raised, including: which of the authors are AI scientists and what is their experience; how were training and test data acquired; what were the input variables; how was the algorithm trained; how was the algorithm evaluated and validated, and was the validation internal or external; are the results reproducible. We believe that one simple solution is to include in the Editorial Board of journals technical reviewers with expertise in AI who are able to ensure the soundness of the technical aspects of a paper and assess interdisciplinary research.

To facilitate reporting of AI trials, the CONSORT (Consolidated Standards of Reporting Trials) and SPIRIT (Standard Protocol Items: Recommendations for Interventional Trials) steering groups are expected to publish the first international consensus‐based reporting guidelines for clinical trials evaluating AI interventions in 2020^69^.

## Summary

AI uses data and algorithms to derive computational models of tasks that are often as good as (or better than) humans. AI is already a part of our daily life and is a prominent source of innovation in healthcare, helping to develop new drugs, support clinical decisions and provide quality assurance. Deep learning performs particularly well in image pattern recognition and solutions based on this approach can benefit healthcare professionals who depend heavily on information obtained from images, such as radiographers, pathologists and sonologists.

We have presented an overview of AI technology and some of the issues related to the introduction of this emerging technology into clinical practice, in the context of ultrasound in obstetrics and gynecology. At this stage, AI applications are in the early stages of deployment and a systematic review would be premature. In addition, performing a clinical systematic review in this area is challenging because most of the published peer‐reviewed scientific articles appear in the engineering literature which usually focuses on the AI methodology and few studies have assessed clinical applicability. Lastly, algorithms and results of approved AI applications are often not published in scientific journals due to commercial sensitivities.

In the past, advances in women's ultrasound have been largely achieved through better imaging, advances in education and training, adherence to guidelines and standards of care, and improvement of genetic technologies^70^. Despite all these advances, the fundamental way in which ultrasound images are acquired and interpreted has remained relatively unchanged. AI opens an opportunity to introduce in the patient–carer relationship a third ‘participant’ that is able to contribute to healthcare. Improved quality through automatic categorization or interpretation of images and ensuring images are fit for purpose can increase confidence in imaging‐based diagnosis. In high‐income settings, this could contribute to healthcare efficiency and workflow improvements in screening. In under‐resourced settings, it opens the prospect of strengthening ultrasound imaging by replicating basic obstetric ultrasound where there is none which could allow, for example, gestational‐age estimation or diagnosis of placenta previa. For this potential to be realized, interdisciplinary communication between AI developers and ultrasound professionals needs to be strengthened. A greater understanding of how AI methods work is important to enable clinicians to trust AI solutions. To ensure seamless integration of AI, medical professional organizations should start considering how AI affects them, recommend that physicians publish their experiences of using AI technologies, and consider appropriate guidelines or committees on aspects of AI.

## Supporting information


**Appendix** **S1** Example: ophthalmology at the forefront of artificial intelligence

**Figure S1** Error rates on ImageNet Large‐Scale Visual Recognition Challenge between 2010 and 2017. Accuracy improved dramatically with introduction of deep learning in 2012 and continued to improve thereafter. Humans perform with an error rate of approximately 5%. Figure reproduced with permission from Langlotz *et al*.^17^.

## References

[1] United Kingdom Engineering and Physical Sciences Research Council . Artificial intelligence technologies. https://epsrc.ukri.org/research/ourportfolio/researchareas/ait/.

[2] Turing AM . I–Computing Machinery and Intelligence. Mind 1950; LIX: 433–460.

[3] McCarthy J , Minsky M , Rochester N , Shannon C. A proposal for the dartmouth summer research project on artificial intelligence, August 1955 http://www‐formal.stanford.edu/jmc/history/dartmouth/dartmouth.html

[4] Bory P . Deep new: The shifting narratives of artificial intelligence from Deep Blue to AlphaGo. Convergence 2019; 25: 627–642.

[5] Abramoff MD , Lavin PT , Birch M , Shah N , Folk JC . Pivotal trial of an autonomous AI‐based diagnostic system for detection of diabetic retinopathy in primary care offices. NPJ Digit Med 2018; 1: 39. 31304320 10.1038/s41746-018-0040-6 PMC6550188

[6] Wang P , Berzin TM , Glissen Brown JR , Bharadwaj S , Becq A , Xiao X , Liu P , Li L , Song Y , Zhang D , Li Y , Xu G , Tu M , Liu X . Real‐time automatic detection system increases colonoscopic polyp and adenoma detection rates: a prospective randomised controlled study. Gut 2019; 68: 1813–1819. 30814121 10.1136/gutjnl-2018-317500 PMC6839720

[7] Rezaii N , Walker E , Wolff P . A machine learning approach to predicting psychosis using semantic density and latent content analysis. NPJ Schizophr 2019; 5: 9. 31197184 10.1038/s41537-019-0077-9 PMC6565626

[8] Artzi NS , Shilo S , Hadar E , Rossman H , Barbash‐Hazan S , Ben‐Haroush A , Balicer RD , Feldman B , Wiznitzer A , Segal E . Prediction of gestational diabetes based on nationwide electronic health records. Nat Med 2020; 26: 71–76. 31932807 10.1038/s41591-019-0724-8

[9] Makino M , Yoshimoto R , Ono M , Itoko T , Katsuki T , Koseki A , Kudo M , Haida K , Kuroda J , Yanagiya R , Saitoh E , Hoshinaga K , Yuzawa Y , Suzuki A . Artificial intelligence predicts the progression of diabetic kidney disease using big data machine learning. Sci Rep 2019; 9: 11862. 31413285 10.1038/s41598-019-48263-5 PMC6694113

[10] Annarumma M , Withey SJ , Bakewell RJ , Pesce E , Goh V , Montana G . Automated Triaging of Adult Chest Radiographs with Deep Artificial Neural Networks. Radiology 2019; 291: 196–202. 30667333 10.1148/radiol.2018180921 PMC6438359

[11] Titano JJ , Badgeley M , Schefflein J , Pain M , Su A , Cai M , Swinburne N , Zech J , Kim J , Bederson J , Mocco J , Drayer B , Lehar J , Cho S , Costa A , Oermann EK . Automated deep‐neural‐network surveillance of cranial images for acute neurologic events. Nat Med 2018; 24: 1337–1341. 30104767 10.1038/s41591-018-0147-y

[12] Liu X , Faes L , Kale AU , Wagner SK , Fu DJ , Bruynseels A , Mahendiran T , Moraes G , Shamdas M , Kern C , Ledsam JR , Schmid MK , Balaskas K , Topol EJ , Bachmann LM , Keane PA , Denniston AK . A comparison of deep learning performance against health‐care professionals in detecting diseases from medical imaging: a systematic review and meta‐analysis. Lancet Digit Health 2019; 1: e271–e297. 10.1016/S2589-7500(19)30123-2 33323251

[13] Marcus GM . The Apple Watch can detect atrial fibrillation: so what now? Nat Rev Cardiol 2020; 17: 135–136. 31873198 10.1038/s41569-019-0330-y

[14] Ianevski A , Giri AK , Gautam P , Kononov A , Potdar S , Saarela J , Wennerberg K , Aittokallio T . Prediction of drug combination effects with a minimal set of experiments. Nat Mach Intell 2019; 1: 568–577. 32368721 10.1038/s42256-019-0122-4 PMC7198051

[15] Wang X , Terashi G , Christoffer CW , Zhu M , Kihara D . Protein Docking Model Evaluation by 3D Deep Convolutional Neural Networks. Bioinformatics 2020; 36: 2113–2118. 31746961 10.1093/bioinformatics/btz870 PMC7141855

[16] Zhdanov A , Atluri S , Wong W , Vaghei Y , Daskalakis ZJ , Blumberger DM , Frey BN , Giacobbe P , Lam RW , Milev R , Mueller DJ , Turecki G , Parikh SV , Rotzinger S , Soares CN , Brenner CA , Vila‐Rodriguez F , McAndrews MP , Kleffner K , Alonso‐Prieto E , Arnott SR , Foster JA , Strother SC , Uher R , Kennedy SH , Farzan F . Use of Machine Learning for Predicting Escitalopram Treatment Outcome From Electroencephalography Recordings in Adult Patients With Depression. JAMA Netw Open 2020; 3: e1918377. 31899530 10.1001/jamanetworkopen.2019.18377 PMC6991244

[17] Langlotz CP , Allen B , Erickson BJ , Kalpathy‐Cramer J , Bigelow K , Cook TS , Flanders AE , Lungren MP , Mendelson DS , Rudie JDJR . A roadmap for foundational research on artificial intelligence in medical imaging: From the 2018 NIH/RSNA/ACR/The Academy Workshop. Radiology 2019; 291: 781–791. 30990384 10.1148/radiol.2019190613 PMC6542624

[18] Safavi KC , Khaniyev T , Copenhaver M , Seelen M , Zenteno Langle AC , Zanger J , Daily B , Levi R , Dunn P . Development and Validation of a Machine Learning Model to Aid Discharge Processes for Inpatient Surgical Care. JAMA Netw Open 2019; 2: e1917221. 31825503 10.1001/jamanetworkopen.2019.17221 PMC6991195

[19] Chang PJ . Moving Artificial Intelligence from Feasible to Real: Time to Drill for Gas and Build Roads. Radiology 2020; 294: 432–433. 31800365 10.1148/radiol.2019192527

[20] Majkowska A , Mittal S , Steiner DF , Reicher JJ , McKinney SM , Duggan GE , Eswaran K , Cameron Chen PH , Liu Y , Kalidindi SR , Ding A , Corrado GS , Tse D , Shetty S . Chest Radiograph Interpretation with Deep Learning Models: Assessment with Radiologist‐adjudicated Reference Standards and Population‐adjusted Evaluation. Radiology 2020; 294: 421–431. 31793848 10.1148/radiol.2019191293

[21] Timmerman D , Verrelst H , Bourne TH , De Moor B , Collins WP , Vergote I , Vandewalle J. Artificial neural network models for the preoperative discrimination between malignant and benign adnexal masses. Ultrasound Obstet Gynecol 1999; 13: 17–25. 10201082 10.1046/j.1469-0705.1999.13010017.x

[22] Eriksson LSE , Epstein E , Testa AC , Fischerova D , Valentin L , Sladkevicius P , Franchi D , Fruhauf F , Fruscio R , Haak LA , Opolskiene G , Mascilini F , Alcazar JL , Van Holsbeke C , Chiappa V , Bourne T , Lindqvist PG , Van Calster B , Timmerman D , Verbakel JY , Van den Bosch T , Wynants L . Ultrasound‐based risk model for preoperative prediction of lymph‐node metastases in women with endometrial cancer: model‐development study. Ultrasound Obstet Gynecol 2020; 56: 443–452. 31840873 10.1002/uog.21950

[23] Huang YL , Chen HY . Computer‐aided diagnosis of urodynamic stress incontinence with vector‐based perineal ultrasound using neural networks. Ultrasound Obstet Gynecol 2007; 30: 1002–1006. 17948232 10.1002/uog.4102

[24] van den Noort F , van der Vaart CH , Grob ATM , van de Waarsenburg MK , Slump CH , van Stralen M . Deep learning enables automatic quantitative assessment of puborectalis muscle and urogenital hiatus in plane of minimal hiatal dimensions. Ultrasound Obstet Gynecol 2019; 54: 270–275. 30461079 10.1002/uog.20181 PMC6772057

[25] Huang YL , Kuo SJ , Chang CS , Liu YK , Moon WK , Chen DR . Image retrieval with principal component analysis for breast cancer diagnosis on various ultrasonic systems. Ultrasound Obstet Gynecol 2005; 26: 558–566. 16086435 10.1002/uog.1951

[26] Kuo SJ , Hsiao YH , Huang YL , Chen DR . Classification of benign and malignant breast tumors using neural networks and three‐dimensional power Doppler ultrasound. Ultrasound Obstet Gynecol 2008; 32: 97–102. 18521971 10.1002/uog.4103

[27] Huang YL , Chen DR , Jiang YR , Kuo SJ , Wu HK , Moon WK . Computer‐aided diagnosis using morphological features for classifying breast lesions on ultrasound. Ultrasound Obstet Gynecol 2008; 32: 565–572. 18383556 10.1002/uog.5205

[28] Neocleous AC , Syngelaki A , Nicolaides KH , Schizas CN . Two‐stage approach for risk estimation of fetal trisomy 21 and other aneuploidies using computational intelligence systems. Ultrasound Obstet Gynecol 2018; 51: 503–508. 28640401 10.1002/uog.17558

[29] Bonet‐Carne E , Palacio M , Cobo T , Perez‐Moreno A , Lopez M , Piraquive JP , Ramirez JC , Botet F , Marques F , Gratacos E. Quantitative ultrasound texture analysis of fetal lungs to predict neonatal respiratory morbidity. Ultrasound Obstet Gynecol 2015; 45: 427–433. 24919442 10.1002/uog.13441

[30] Bahado‐Singh RO , Sonek J , McKenna D , Cool D , Aydas B , Turkoglu O , Bjorndahl T , Mandal R , Wishart D , Friedman P , Graham SF , Yilmaz A. Artificial intelligence and amniotic fluid multiomics: prediction of perinatal outcome in asymptomatic women with short cervix. Ultrasound Obstet Gynecol 2019; 54: 110–118. 30381856 10.1002/uog.20168

[31] Tsur A , Batsry L , Toussia‐Cohen S , Rosenstein MG , Barak O , Brezinov Y , Yoeli‐Ullman R , Sivan E , Sirota M , Druzin ML , Stevenson DK , Blumenfeld YJ , Aran D . Development and validation of a machine‐learning model for prediction of shoulder dystocia. Ultrasound Obstet Gynecol 2020; 56: 588–596. 31587401 10.1002/uog.21878

[32] Jugovic D , Tumbri J , Medic M , Jukic MK , Kurjak A , Arbeille P , Salihagic‐Kadic A . New Doppler index for prediction of perinatal brain damage in growth‐restricted and hypoxic fetuses. Ultrasound Obstet Gynecol 2007; 30: 303–311. 17721870 10.1002/uog.4094

[33] Papageorghiou AT , Kemp B , Stones W , Ohuma EO , Kennedy SH , Purwar M , Salomon LJ , Altman DG , Noble JA , Bertino E , Gravett MG , Pang R , Cheikh Ismail L , Barros FC , Lambert A , Jaffer YA , Victora CG , Bhutta ZA , Villar J , International Fetal and Newborn Growth Consortium for the 21st Century (INTERGROWTH‐21st) . Ultrasound‐based gestational‐age estimation in late pregnancy. Ultrasound Obstet Gynecol 2016; 48: 719–726. 26924421 10.1002/uog.15894 PMC6680349

[34] Xie HN , Wang N , He M , Zhang LH , Cai HM , Xian JB , Lin MF , Zheng J , Yang YZ . Using deep‐learning algorithms to classify fetal brain ultrasound images as normal or abnormal. Ultrasound Obstet Gynecol 2020; 56: 579–587. 31909548 10.1002/uog.21967

[35] Gobet F. The Future of Expertise: The Need for a Multidisciplinary Approach. Journal of Expertise 2018; 1: 107–113.

[36] Littmann M , Selig K , Cohen‐Lavi L , Frank Y , Hönigschmid P , Kataka E , Mösch A , Qian K , Ron A , Schmid S , Sorbie A , Szlak L , Dagan‐Wiener A , Ben‐Tal N , Niv MY , Razansky D , Schuller BW , Ankerst D , Hertz T , Rost B . Validity of machine learning in biology and medicine increased through collaborations across fields of expertise. Nat Mach Intell 2020; 2: 18–24.

[37] American College of Radiology Data Science Institute . FDA Cleared AI Algorithms. https://www.acrdsi.org/DSI‐Services/FDA‐Cleared‐AI‐Algorithms [Accessed May 7th, 2020].

[38] Food and Drug Administration . FDA Authorizes Marketing of First Cardiac Ultrasound Software That Uses Artificial Intelligence to Guide User https://www.fda.gov/news‐events/press‐announcements/fda‐authorizes‐marketing‐first‐cardiac‐ultrasound‐software‐uses‐artificial‐intelligence‐guide‐user. 2020.

[39] Harris S. Signify Research. Artificial Inelligence in Medical Imaging to Top $2 Billion by 2023. https://www.signifyresearch.net/medical‐imaging/ai‐medical‐imaging‐top‐2‐billion‐2023/ [Accessed March 2nd, 2020].

[40] Ghorbani A , Ouyang D , Abid A , He B , Chen JH , Harrington RA , Liang DH , Ashley EA , Zou JY . Deep learning interpretation of echocardiograms. NPJ Digit Med 2020; 3: 10. 31993508 10.1038/s41746-019-0216-8 PMC6981156

[41] Liu S , Wang Y , Yang X , Lei B , Liu L , Li SX , Ni D , Wang T . Deep Learning in Medical Ultrasound Analysis: A Review. Engineering 2019; 5: 261–275.

[42] Ouyang D , He B , Ghorbani A , Yuan N , Ebinger J , Langlotz CP , Heidenreich PA , Harrington RA , Liang DH , Ashley EA , Zou JY . Video‐based AI for beat‐to‐beat assessment of cardiac function. Nature 2020; 580: 252–256. 32269341 10.1038/s41586-020-2145-8 PMC8979576

[43] Sharma H , Droste R , Chatelain P , Drukker L , Papageorghiou AT , Noble JA . Spatio‐Temporal Partitioning And Description Of Full‐Length Routine Fetal Anomaly Ultrasound Scans. Proc IEEE Int Symp Biomed Imaging 2019; 16: 987–990. 31993109 10.1109/ISBI.2019.8759149 PMC6986911

[44] Baumgartner CF , Kamnitsas K , Matthew J , Smith S , Kainz B , Rueckert D . Real‐Time Standard Scan Plane Detection and Localisation in Fetal Ultrasound Using Fully Convolutional Neural Networks In Medical Image Computing and Computer‐Assisted Intervention – MICCAI 2016. Ourselin S, Joskowicz L, Sabuncu MR, Unal G, Wells W (eds). Springer International Publishing: Cham, 2016; 203–211.

[45] Chen H , Wu L , Dou Q , Qin J , Li S , Cheng J , Ni D , Heng P . Ultrasound Standard Plane Detection Using a Composite Neural Network Framework. IEEE Trans Cybern 2017; 47: 1576–1586. 28371793 10.1109/TCYB.2017.2685080

[46] Singhal N , Mukherjee S , Perrey C . Automated assessment of endometrium from transvaginal ultrasound using Deep Learned Snake. Presented at 2017 IEEE 14th International Symposium on Biomedical Imaging, 2017; 283–286.

[47] Allen B , Dreyer K , McGinty GD . Integrating Artificial Intelligence Into Radiologic Practice: A Look to the Future. J Am Coll Radiol 2020; 17: 280–283. 31790672 10.1016/j.jacr.2019.10.010

[48] Frank MR , Autor D , Bessen JE , Brynjolfsson E , Cebrian M , Deming DJ , Feldman M , Groh M , Lobo J , Moro E , Wang D , Youn H , Rahwan I . Toward understanding the impact of artificial intelligence on labor. Proc Natl Acad Sci USA 2019; 116: 6531–6539. 30910965 10.1073/pnas.1900949116 PMC6452673

[49] Topol EJ . Chapter six: Doctors and Patterns In: Deep medicine: how artificial intelligence can make healthcare human again (first edn). Basic Books: New York, 2019; 111–135.

[50] Recht M , Bryan RN . Artificial Intelligence: Threat or Boon to Radiologists? J Am Coll Radiol 2017; 14: 1476–1480. 28826960 10.1016/j.jacr.2017.07.007

[51] Mazurowski MA . Artificial Intelligence May Cause a Significant Disruption to the Radiology Workforce. J Am Coll Radiol 2019; 16: 1077–1082. 30975611 10.1016/j.jacr.2019.01.026

[52] Noseworthy J . The Future of Care ‐ Preserving the Patient‐Physician Relationship. N Engl J Med 2019; 381: 2265–2269. 31800995 10.1056/NEJMsr1912662

[53] Waring L , Miller PK , Sloane C , Bolton G . Charting the practical dimensions of understaffing from a managerial perspective: The everyday shape of the UK's sonographer shortage. Ultrasound 2018; 26: 206–213. 30479635 10.1177/1742271X18772606 PMC6243452

[54] Nelson CA , Butte AJ , Baranzini SE . Integrating biomedical research and electronic health records to create knowledge‐based biologically meaningful machine‐readable embeddings. Nat Commun 2019; 10: 3045. 31292438 10.1038/s41467-019-11069-0 PMC6620318

[55] Kansagra AP , Yu JP , Chatterjee AR , Lenchik L , Chow DS , Prater AB , Yeh J , Doshi AM , Hawkins CM , Heilbrun ME , Smith SE , Oselkin M , Gupta P , Ali S . Big Data and the Future of Radiology Informatics. Acad Radiol 2016; 23: 30–42. 26683510 10.1016/j.acra.2015.10.004

[56] Chiappa V , Bogani G , Ditto A , Martinelli F , Murru G , Raspagliesi F. OP07.10: Artificial intelligence weights the importance of clinical and sonographic factors predicting nodal metastasis in endometrial cancer. Ultrasound Obstet Gynecol 2019; 54 **(** S1 **)**: 107–107.

[57] Babic B , Gerke S , Evgeniou T , Cohen IG . Algorithms on regulatory lockdown in medicine. Science 2019; 366: 1202–1204. 31806804 10.1126/science.aay9547

[58] Wiens J , Price WN , Sjoding MW . Diagnosing bias in data‐driven algorithms for healthcare. Nat Med 2020; 26: 25–26. 31932798 10.1038/s41591-019-0726-6

[59] Drukker L , Droste R , Chatelain P , Noble JA , Papageorghiou AT . Expected‐value bias in routine third‐trimester growth scans. Ultrasound Obstet Gynecol 2020; 55: 375–382. 31763735 10.1002/uog.21929 PMC7079033

[60] Nagendran M , Chen Y , Lovejoy CA , Gordon AC , Komorowski M , Harvey H , Topol EJ , Ioannidis JPA , Collins GS , Maruthappu M . Artificial intelligence versus clinicians: systematic review of design, reporting standards, and claims of deep learning studies. BMJ 2020; 368: m689. 32213531 10.1136/bmj.m689 PMC7190037

[61] Brundage M , Avin S , Wang J , Belfield H , Krueger G , Hadfield G , Khlaaf H , Yang J , Toner H , Fong R , Maharaj T , Koh PW , Hooker S , Leung J , Trask A , Bluemke E , Lebensold J , O'Keefe C , Koren M , Ryffel T , Rubinovitz JB , Besiroglu T , Carugati F , Clark J , Eckersley P , de Haas S , Johnson M , Laurie B , Ingerman A , Krawczuk I , Askell A , Cammarota R , Lohn A , Krueger D , Stix C , Henderson P , Graham L , Prunkl C , Martin B , Seger E , Zilberman N , Ó hÉigeartaigh S , Kroeger F , Sastry G , Kagan R , Weller A , Tse B , Barnes E , Dafoe A , Scharre P , Herbert‐Voss A , Rasser M , Sodhani S , Flynn C , Gilbert TK , Dyer L , Khan S , Bengio Y , Anderljung M . *Toward Trustworthy AI Development: Mechanisms for Supporting Verifiable Claims*. April 2020 https://arxiv.org/pdf/2004.07213.pdf

[62] Institute for Ethics in AI. University of Oxford. https://www.schwarzmancentre.ox.ac.uk/ethicsinai [Accessed May 7th, 2020].

[63] Awad E , Dsouza S , Kim R , Schulz J , Henrich J , Shariff A , Bonnefon JF , Rahwan I . The Moral Machine experiment. Nature 2018; 563: 59–64. 30356211 10.1038/s41586-018-0637-6

[64] Char DS , Shah NH , Magnus D . Implementing Machine Learning in Health Care ‐ Addressing Ethical Challenges. N Engl J Med 2018; 378: 981–983. 29539284 10.1056/NEJMp1714229 PMC5962261

[65] Mittelstadt B . Principles alone cannot guarantee ethical AI. Nat Mach Intell 2019; 1: 501–507.

[66] Geis JR , Brady AP , Wu CC , Spencer J , Ranschaert E , Jaremko JL , Langer SG , Borondy Kitts A , Birch J , Shields WF , van den Hoven van Genderen R , Kotter E , Wawira Gichoya J , Cook TS , Morgan MB , Tang A , Safdar NM , Kohli M . Ethics of Artificial Intelligence in Radiology: Summary of the Joint European and North American Multisociety Statement. Radiology 2019; 293: 436–440. 31573399 10.1148/radiol.2019191586

[67] Price WN 2nd , Gerke S , Cohen IG . Potential Liability for Physicians Using Artificial Intelligence. JAMA 2019 DOI: 10.1001/jama.2019.15064. 31584609

[68] Liu Y , Chen PC , Krause J , Peng L . How to Read Articles That Use Machine Learning: Users' Guides to the Medical Literature. JAMA 2019; 322: 1806–1816. 31714992 10.1001/jama.2019.16489

[69] CONSORT‐AI and SPIRIT‐AI Steering Group . Reporting guidelines for clinical trials evaluating artificial intelligence interventions are needed. Nat Med 2019; 25: 1467–1468. 31551578 10.1038/s41591-019-0603-3

[70] Abu‐Rustum RS , Abuhamad AZ . Fetal imaging: past, present, and future. A journey of marvel. BJOG 2018; 125: 1568. 30302930 10.1111/1471-0528.15343

[71] The Alan Turing Institute . Frequently Asked Questions. https://www.turing.ac.uk/about‐us/frequently‐asked‐questions [Accessed December 20th, 2019].

