# Supplementary material for: Introduction to artificial intelligence in ultrasound imaging in obstetrics and gynecology
Source: Ultrasound Obstet Gynecol. 2020 Oct 1;56(4):498–505. doi: 10.1002/uog.22122 (PMC7702141; doi:10.1002/uog.22122)
Supplement: Supplementary file 1 — Appendix S1 Example: ophthalmology at the forefront of artificial intelligence [file UOG-56-498-s001.docx]

**Appendix S1 Example: ophthalmology at the forefront of artificial intelligence**

Automated Retinal Disease Assessment (ARDA) is an excellent example of how AI aims to transform healthcare^1^.

*Background*

Retinal OCT (Optical Coherence Tomography) is an optical, non-invasive, widely performed scan for comprehensive initial assessment and triage of patients with acute and chronic sight loss. This scan provides a 3D volumetric image of the retina that requires an expert to interpret (despite the similar names, OCT and CT use different technologies). With millions^1^ of scans performed annually, a delay between scan and referral for treatment may arise, which can ultimately result in vision loss.

*The AI application*

ARDA is an AI application that instantly interprets OCT scans from routine clinical practice and recommends the urgency of patient referral for treatment for over 50 sight-threatening eye diseases. This application was created by Google DeepMind Health engineers and ophthalmologists and is as accurate as expert doctors.

*How was the application developed? (simplified)*

A *training dataset* was created from 14,884 OCT scans of patients who were referred to hospital with symptoms suggestive of macular pathology. For each OCT scan, individual diagnoses and treatment information recorded in the notes were used to retrospectively *label* each scan with a specific triage decision: urgent referral, semi-urgent referral, routine referral, and observation only. Next, the training dataset was used to train deep learning convolutional neural networks.

Thereafter, a *testing dataset* of 997 randomly selected unlabeled OCT scans (from patients not included in the training dataset) were used to evaluate the application. These testing OCT scans were reviewed by the ARDA application and a group of experts (four ophthalmologists-retina experts with 12-21 years of clinical experience and four optometrists with OCT specialty training and 3-15 years of clinical experience).

Finally, the agreement between experts and the application was tested. The results show that the application performs on par with retina expert ophthalmologists and better than retina expert optometrists for the triaging of retinal disease from OCT scans. This was achieved with similar false-positive rates, meaning that the application did not increase the rate of unnecessary referrals.

*Limitations*

The limitations of the applications should also be noted and include the testing on specific OCT machines and the exclusion of low-quality OCTs or those showing extremely rare diagnoses. This means that it’s difficult to know how the application will perform when introduced with OCT scans from a new machine model, of low-quality, or showing a rare disease.

*Other examples*

Many similar studies have demonstrated even or superior AI performance in other medical image analysis tasks, including mammography breast cancer screening^2^, prostate cancer biopsy^3^, low-dose chest computerized tomography lung cancer screening^4^, and skin lesion image for skin cancer classification^5^.

**References:**

1. De Fauw J, Ledsam JR, Romera-Paredes B, Nikolov S, Tomasev N, Blackwell S, Askham H, Glorot X, O'Donoghue B, Visentin D, van den Driessche G, Lakshminarayanan B, Meyer C, Mackinder F, Bouton S, Ayoub K, Chopra R, King D, Karthikesalingam A, Hughes CO, Raine R, Hughes J, Sim DA, Egan C, Tufail A, Montgomery H, Hassabis D, Rees G, Back T, Khaw PT, Suleyman M, Cornebise J, Keane PA, Ronneberger O. Clinically applicable deep learning for diagnosis and referral in retinal disease. *Nat Med* 2018; **24**: 1342–1350.
2. McKinney SM, Sieniek M, Godbole V, Godwin J, Antropova N, Ashrafian H, Back T, Chesus M, Corrado GC, Darzi A, Etemadi M, Garcia-Vicente F, Gilbert FJ, Halling-Brown M, Hassabis D, Jansen S, Karthikesalingam A, Kelly CJ, King D, Ledsam JR, Melnick D, Mostofi H, Peng L, Reicher JJ, Romera-Paredes B, Sidebottom R, Suleyman M, Tse D, Young KC, De Fauw J, Shetty S. International evaluation of an AI system for breast cancer screening. *Nature* 2020; **577**: 89–94.
3. Ström P, Kartasalo K, Olsson H, Solorzano L, Delahunt B, Berney DM, Bostwick DG, Evans AJ, Grignon DJ, Humphrey PA. Artificial intelligence for diagnosis and grading of prostate cancer in biopsies: a population-based, diagnostic study. *Lancet Oncol* 2020; **21**: 222–232.
4. Ardila D, Kiraly AP, Bharadwaj S, Choi B, Reicher JJ, Peng L, Tse D, Etemadi M, Ye W, Corrado G, Naidich DP, Shetty S. End-to-end lung cancer screening with three-dimensional deep learning on low-dose chest computed tomography. *Nat Med* 2019; **25**: 954–961.
5. Esteva A, Kuprel B, Novoa RA, Ko J, Swetter SM, Blau HM, Thrun S. Dermatologist-level classification of skin cancer with deep neural networks. *Nature* 2017; **542**: 115–118.
