# Supplementary figures and images for: Introduction to artificial intelligence in ultrasound imaging in obstetrics and gynecology
Source: Ultrasound Obstet Gynecol. 2020 Oct 1;56(4):498–505. doi: 10.1002/uog.22122 (PMC7702141; doi:10.1002/uog.22122)

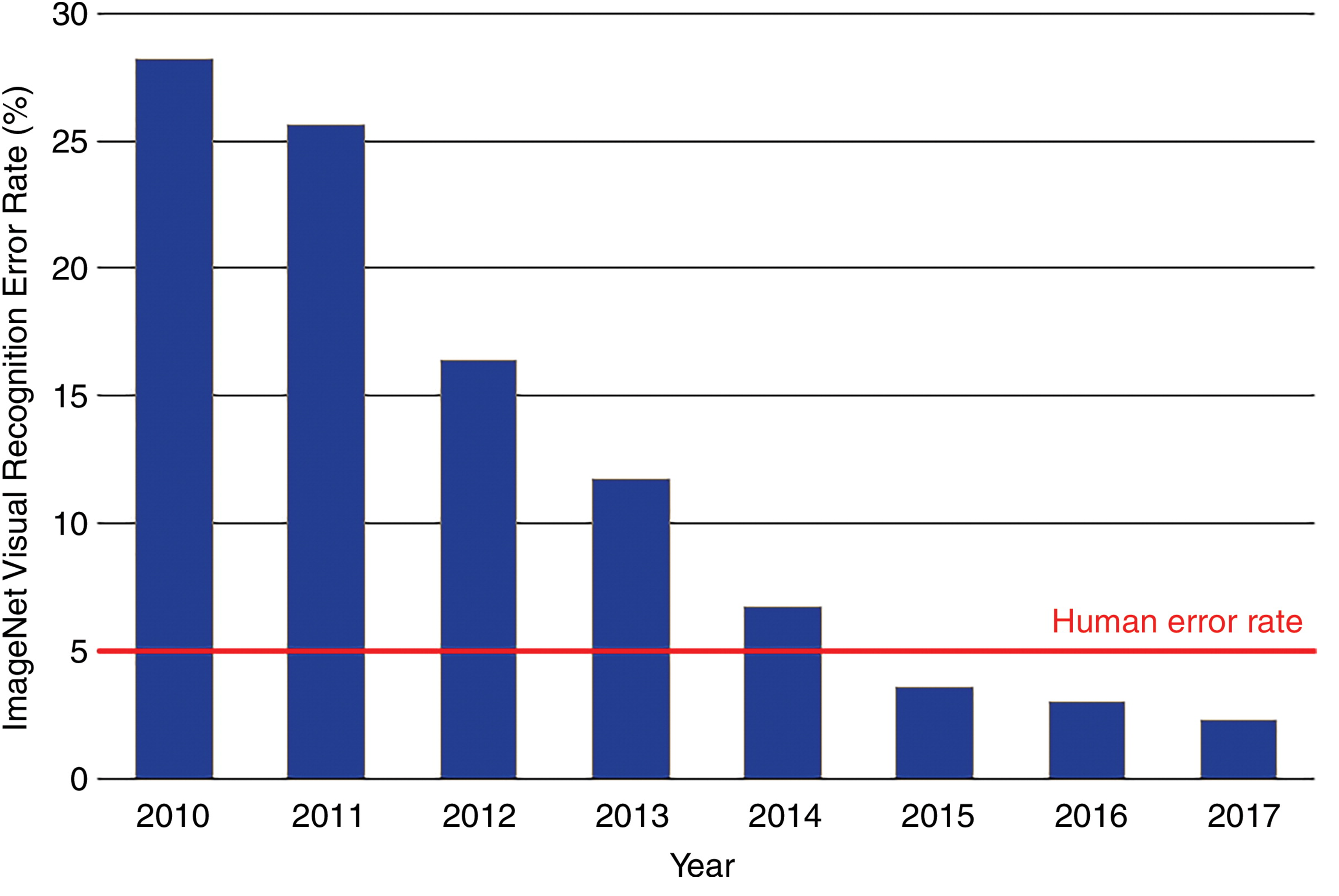

Supplement: Supplementary file 2 — Figure S1 Error rates on ImageNet Large‐Scale Visual Recognition Challenge between 2010 and 2017. Accuracy improved dramatically with introduction of deep learning in 2012 and continued to improve thereafter. Humans perform with an error rate of approximately 5%. Figure reproduced with permission from Langlotz et al. 17 . [file UOG-56-498-s002.TIF]
